# Supplementary material for: Preserving ester-linked modifications reveals glutamate and aspartate mono-ADP-ribosylation by PARP1 and its reversal by PARG
Source: Nat Commun. 2024 May 18;15:4239. doi: 10.1038/s41467-024-48314-0 (PMC11102441; doi:10.1038/s41467-024-48314-0)
Supplement: Supplementary file 1 — Supplementary Information [file 41467_2024_48314_MOESM1_ESM.pdf]

SUPPLEMENTARY INFORMATION

Preserving Ester-linked Modifications Reveals Glutamate and Aspartate mono-ADP-ribosylation by PARP1 and its Reversal by PARG

Longarini and Matic

Supplementary Figures 1 – 7

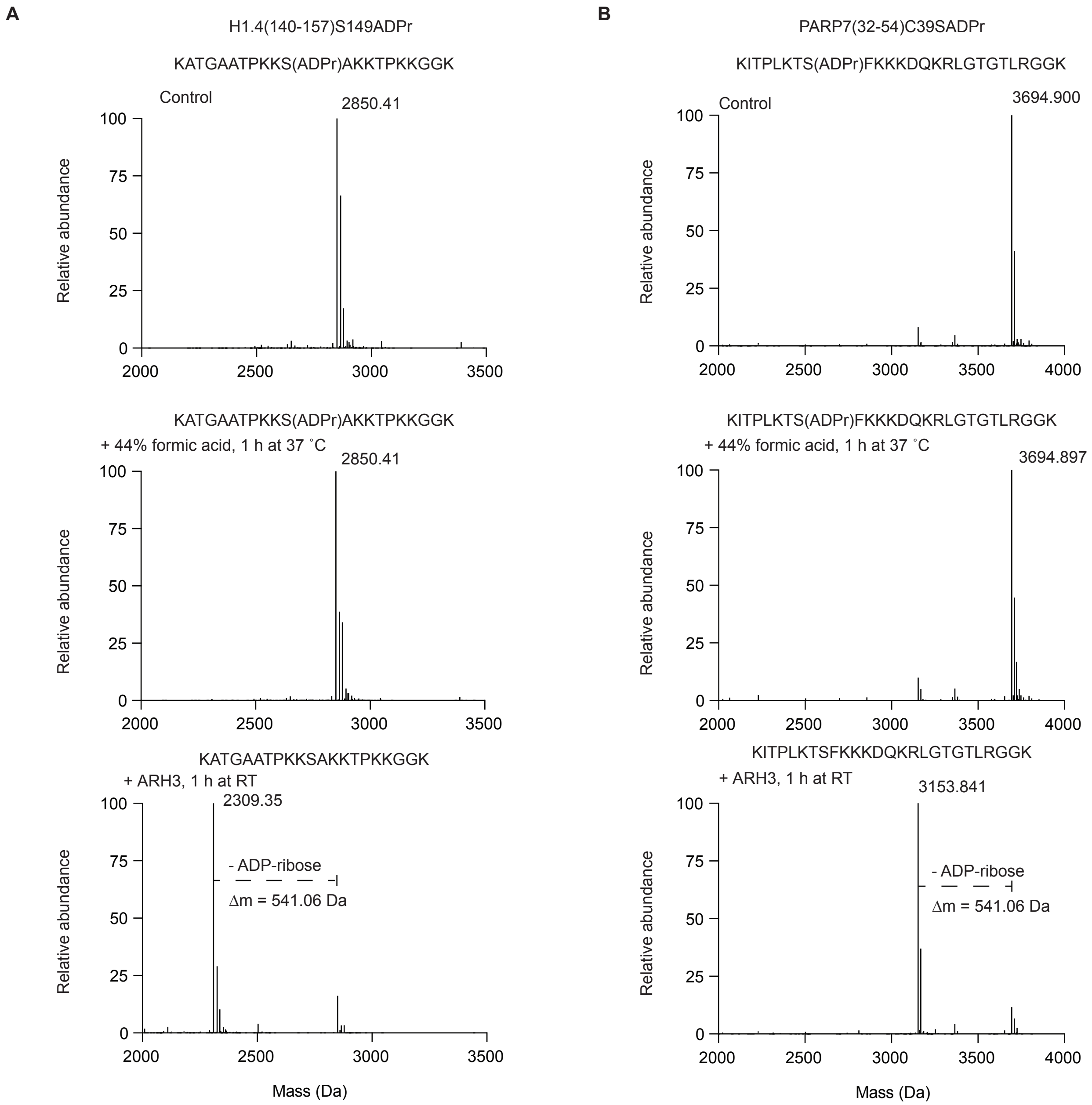
A
H1.4(140-157)S149ADPr
KATGAATPKKS(ADPr)AKKTPKKGGK
Control
2850.41
KATGAATPKKS(ADPr)AKKTPKKGGK
+ 44% formic acid, 1 h at 37 °C
2850.41
KATGAATPKKSAKKTPKKGGK
+ ARH3, 1 h at RT
2309.35
- ADP-ribose
Δm = 541.06 Da
Relative abundance
Mass (Da)
B
PARP7(32-54)C39SADPr
KITPLKTS(ADPr)FKKKDQKRLGTGTLRGGK
Control
3694.900
KITPLKTS(ADPr)FKKKDQKRLGTGTLRGGK
+ 44% formic acid, 1 h at 37 °C
3694.897
KITPLKTSFKKKDQKRLGTGTLRGGK
+ ARH3, 1 h at RT
3153.841
- ADP-ribose
Δm = 541.06 Da
Relative abundance
Mass (Da)

Supp. Fig. 1: The high chemical stability of serine ADP-ribosylation.

a, Deconvoluted MS spectra of biotinylated Histone H1.4 (140-157) S149 ADPribosylated peptide untreated (top), treated with 44 % formic acid in water for 1 h at 37 ˚C (middle) or treated with recombinant ARH3 for 1 h at RT (bottom). As depicted, the *in vitro* ARH3 reaction produces a mass shift of 541.06 Da, corresponding to the loss of ADPr. Conversely, no appreciable loss of ADPr is observed in the formic acid treated sample. **b,** Deconvoluted MS spectra of biotinylated PARP7 (32-54) C39S ADPribosylated peptide untreated (top), treated with 44 % formic acid in water for 1 h at 37 ˚C (middle) or treated with recombinant ARH3 for 1 h at RT (bottom). As depicted, the *in vitro* ARH3 reaction produces a mass shift of 541.06 Da, corresponding to the loss of ADPr. Conversely, no appreciable loss of ADPr is observed in the formic acid treated sample.

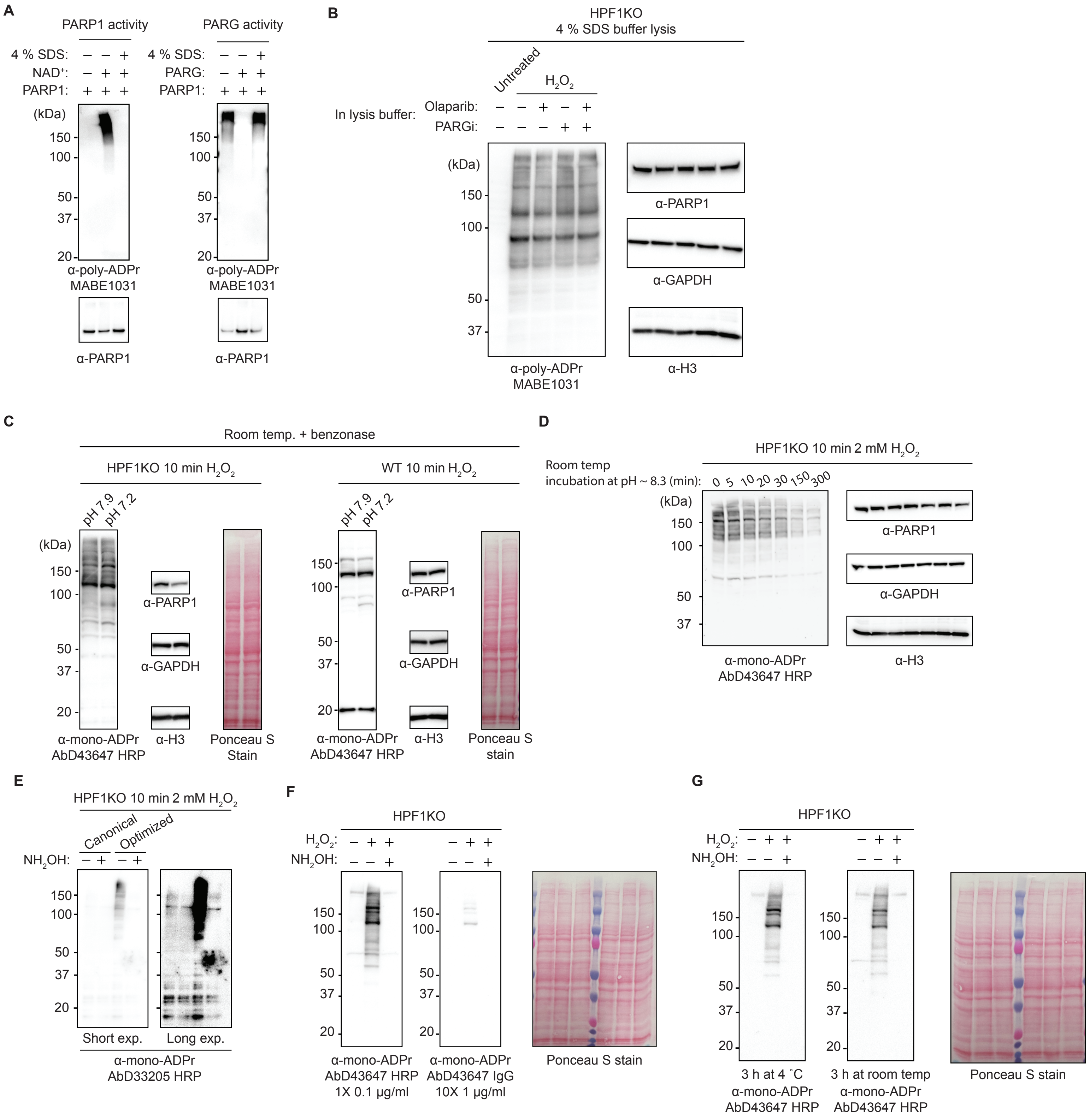
A
PARP1 activity
PARG activity
4 % SDS: – – +
NAD⁺: – + +
PARP1: + + +
4 % SDS: – – +
PARG: – + +
PARP1: + + +
(kDa)
150
100
50
37
20
α-poly-ADPr MABE1031
α-PARP1
B
HPF1KO
4 % SDS buffer lysis
Untreated
H2O2
In lysis buffer:
Olaparib: – – + – +
PARGi: – – – + +
(kDa)
150
100
50
37
α-poly-ADPr MABE1031
α-PARP1
α-GAPDH
α-H3
C
Room temp. + benzonase
HPF1KO 10 min H2O2
WT 10 min H2O2
pH 7.9
pH 7.2
(kDa)
150
100
50
37
20
α-mono-ADPr AbD43647 HRP
α-PARP1
α-GAPDH
α-H3
Ponceau S Stain
Ponceau S stain
D
HPF1KO 10 min 2 mM H2O2
Room temp incubation at pH ~ 8.3 (min): 0 5 10 20 30 150 300
(kDa)
150
100
50
37
α-mono-ADPr AbD43647 HRP
α-PARP1
α-GAPDH
α-H3
E
HPF1KO 10 min 2 mM H2O2
Canonical
Optimized
NH2OH: – + – +
150
100
50
37
20
Short exp.
Long exp.
α-mono-ADPr AbD33205 HRP
F
HPF1KO
H2O2: – + +
NH2OH: – – +
150
100
50
37
20
α-mono-ADPr AbD43647 HRP 1X 0.1 µg/ml
α-mono-ADPr AbD43647 IgG 10X 1 µg/ml
Ponceau S stain
G
HPF1KO
H2O2: – + +
NH2OH: – – +
150
100
50
37
20
3 h at 4 °C α-mono-ADPr AbD43647 HRP
3 h at room temp α-mono-ADPr AbD43647 HRP
Ponceau S stain

Supp. Fig. 2: Preservation of ester-linked modifications reveals DNA damage-induced mono-Asp/Glu-ADPr.

a, Immunoblotting images showing the effect of the denaturing agent SDS on recombinant PARP1 and PARG. Left: PARP1 was automodified as described in the Methods and incubated in the presence or absence of 4% SDS for 30 min, then immunoblotted with the indicated antibodies. Right: Automodified PARP1, inhibited by Olaparib, was incubated with PARG and 4 % SDS, as indicated, for 30 minutes. Shown a representative of two independent experiments. **b**, HPF1KO cells were treated with H_2O_2, then harvested and lysed at room temperature with lysis buffer containing 4 % SDS and, where indicated, PARP1 and PARG inhibitors. Shown is a representative of two independent experiments. **c,** Immunoblotting images showing the effect of pH during sample processing on mono-ADPr signal. HPF1KO U2OS cells were treated with 2 mM H_2O_2 for 10 min, followed by harvesting and lysis at pH 7.9 or 7.2 (4 % SDS, 50 mM HEPES pH 7.2 or 7.9, 150 mM NaCl, 5 mM $MgCl_2$), processed at room temperature and treated with benzonase, followed by immunoblotting with the indicated antibodies and stained with Ponceau S to show total protein load. Shown is a representative of four independent experiments. **d,** Immunoblotting images showing the effect of pH upon addition of 1X NuPAGE LDS sample loading buffer (4X NuPAGE buffer has a pH of 8.5, resulting in a final sample pH of approximately 8.3 when added as 1X final concentration in pH 7.2 lysis buffer) and incubation at room temperature for the indicated times before loading for SDS-PAGE gel. Shown is a representative of three independent experiments. **e,** Related to Fig. 2c, the same samples were also stained with the AbD33205 HRP anti-mono-ADPr antibody, shown here. **f,** Immunoblotting images showing comparison between IgG and HRP-coupled formats for the AbD43647 antibody. 10-fold higher amounts of IgG antibody (1 µg/ml for IgG format and 0.1 µg/ml for HRP format, MW for both at approx. 200 kDa). Both membranes derive from the same samples and were processed and imaged together, with the exception of the antibody incubation step. **g,** HPF1KO U2OS samples were treated and immunoblotted as indicated. After overnight incubation with AbD43647, the membrane was washed in PBS-T, then cut in two. One membrane was further incubated for 3 h at room temp. in PBS-T, while the other membrane was kept at 4 ˚C for the same amount of time. Following incubation, both membranes were imaged together. Both membranes derive from the same samples and were processed together with the exception of the antibody incubation step. For **f, g** shown is a representative of two independent experiments.

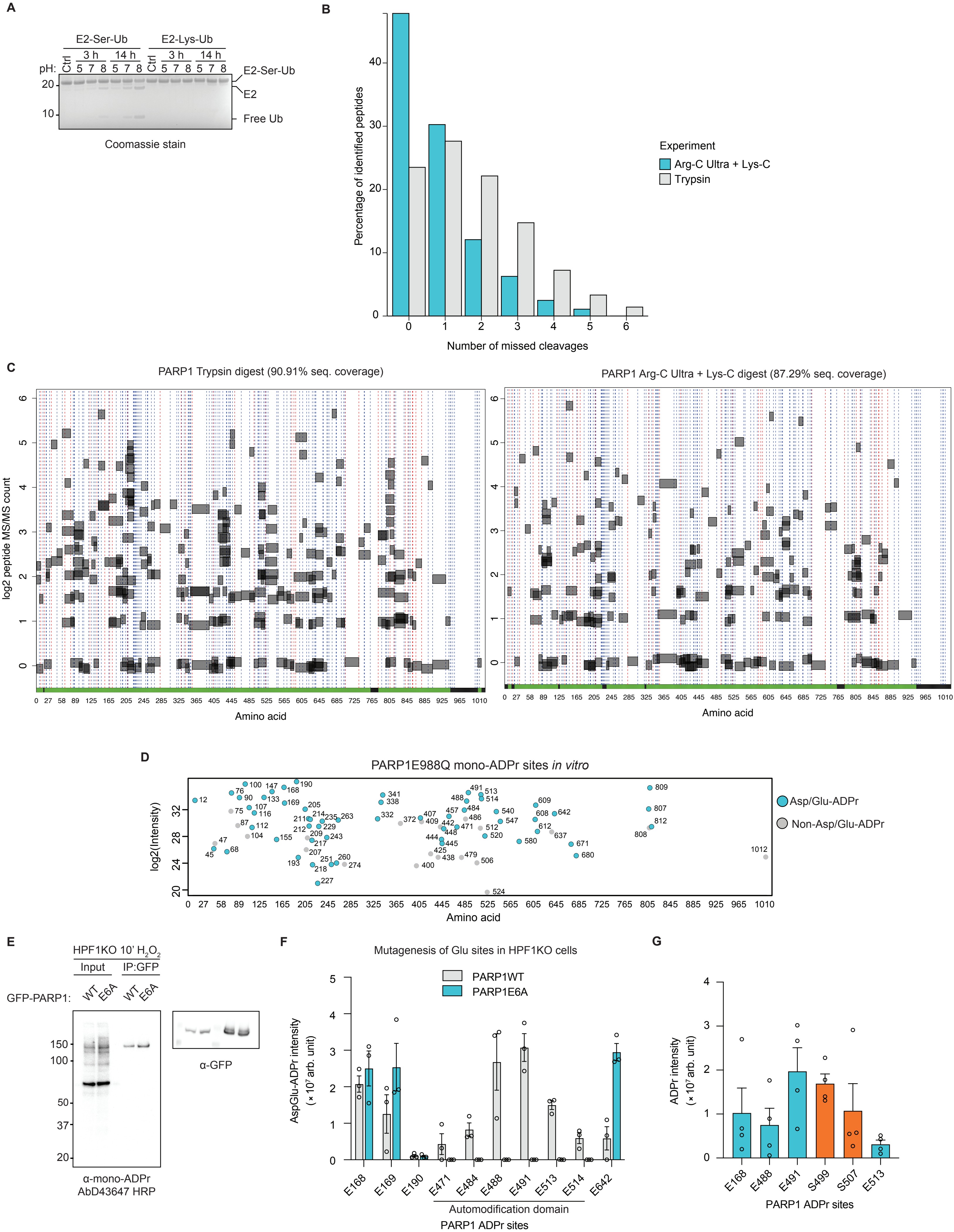
A
E2-Ser-Ub
E2-Lys-Ub
3 h
14 h
pH:
Ctrl
5 7 8
20
10
E2-Ser-Ub
E2
Free Ub
Coomassie stain
B
Percentage of identified peptides
Number of missed cleavages
Experiment
Arg-C Ultra + Lys-C
Trypsin
C
PARP1 Trypsin digest (90.91% seq. coverage)
PARP1 Arg-C Ultra + Lys-C digest (87.29% seq. coverage)
log2 peptide MS/MS count
Amino acid
D
PARP1E988Q mono-ADPr sites *in vitro*
log2(Intensity)
Amino acid
Asp/Glu-ADPr
Non-Asp/Glu-ADPr
E
HPF1KO 10' H_2O_2
Input
IP:GFP
GFP-PARP1:
WT
E6A
150
100
50
37
20
α-mono-ADPr
AbD43647 HRP
α-GFP
F
Mutagenesis of Glu sites in HPF1KO cells
PARP1WT
PARP1E6A
AspGlu-ADPr intensity
(× 10^7 arb. unit)
E168
E169
E190
E471
E484
E488
E491
E513
E514
E642
Automodification domain
PARP1 ADPr sites
G
ADPr intensity
(× 10^7 arb. unit)
E168
E488
E491
S499
S507
E513
PARP1 ADPr sites

Supp. Fig. 3: Short acidic protein digestion with Arg-C Ultra preserves mono-Asp/GluADPr for proteomic analyses.

a, SDS-PAGE showing hydrolysis of serine-linked ubiquitination across a spectrum of incubation conditions for protease digestion. Recombinant UBE2D3 S22R C85S (E2) is linked to the C-terminus of ubiquitin (Ub) with the C85S residue. UBE2D3 S22R C85K linked to the C-term of Ub with a canonical isopeptide bond was used as a control. Each E2~Ub construct was incubated at 37℃ for 3 h or 14 h at pH 5 (20 mM sodium acetate), pH 7 (20 mM HEPES), or pH 8 (20 mM TRIS-HCl), run on SDS-PAGE gel and visualized with Coomassie stain. Shown is a representative of two independent experiments. **b,** percentage of missed cleavages from LC-MS/MS analyses of recombinant PARP1E988Q samples digested with standard "Trypsin" or with our optimized "Arg-C Ultra + Lys-C" protocol (see Fig. 3a). **c,** Schematic illustration of PARP1E988Q sequence coverage. Left: "Trypsin" processed sample, right: "Arg-C Ultra + Lys-C" sample. Each grey bar represents an identified peptide along the corresponding PARP1 sequence (x-axis) and at a given number of MS/MS spectral counts (y-axis). **d,** Recombinant PARP1E988Q was automodified and then digested with the optimized digestion protocol, followed by mass-spectrometry analysis. The scatterplot depicts the $\log_2$ Intensity of identified Asp/Glu-ADPr sites (in cyan) or non-Asp/Glu-ADPr sites (in grey) plotted as a function of their relative position across the PARP1 sequence. **e,** Immunoblotting images of GFP-PARP1 pulldown. WT or E6A (E471A, E484A, E491A, E513A, E514A) PARP1 was immunoprecipitated with a stringent protocol involving urea washes that generates a highly pure eluate, then immunoblotted with the indicated antibodies. **f,** As in Supplementary Fig. 3e, GFP-PARP1 WT or E6A mutant was immunoprecipitated with a highly stringent protocol, digested with the optimized protocol, and subjected to mass-spectrometry analysis. Bar plot ± SEM of the ADPr intensity of each modified site, showing that while in the PARP1E6A mutant there is no automodification on the E471A, E484A, E491A, E513A, E514A sites, this is accompained by a corresponding increase in ADPr status of E168, E169 and E642. n = 3 independent replicates shown as individual dots. **g,** Bar plot ± SEM of the ADPr intensity of each modified site, from GFP-PARP1 pulldown in WT cells treated with H_2O_2 for 10 minutes. n = 4 independent replicates shown as individual dots

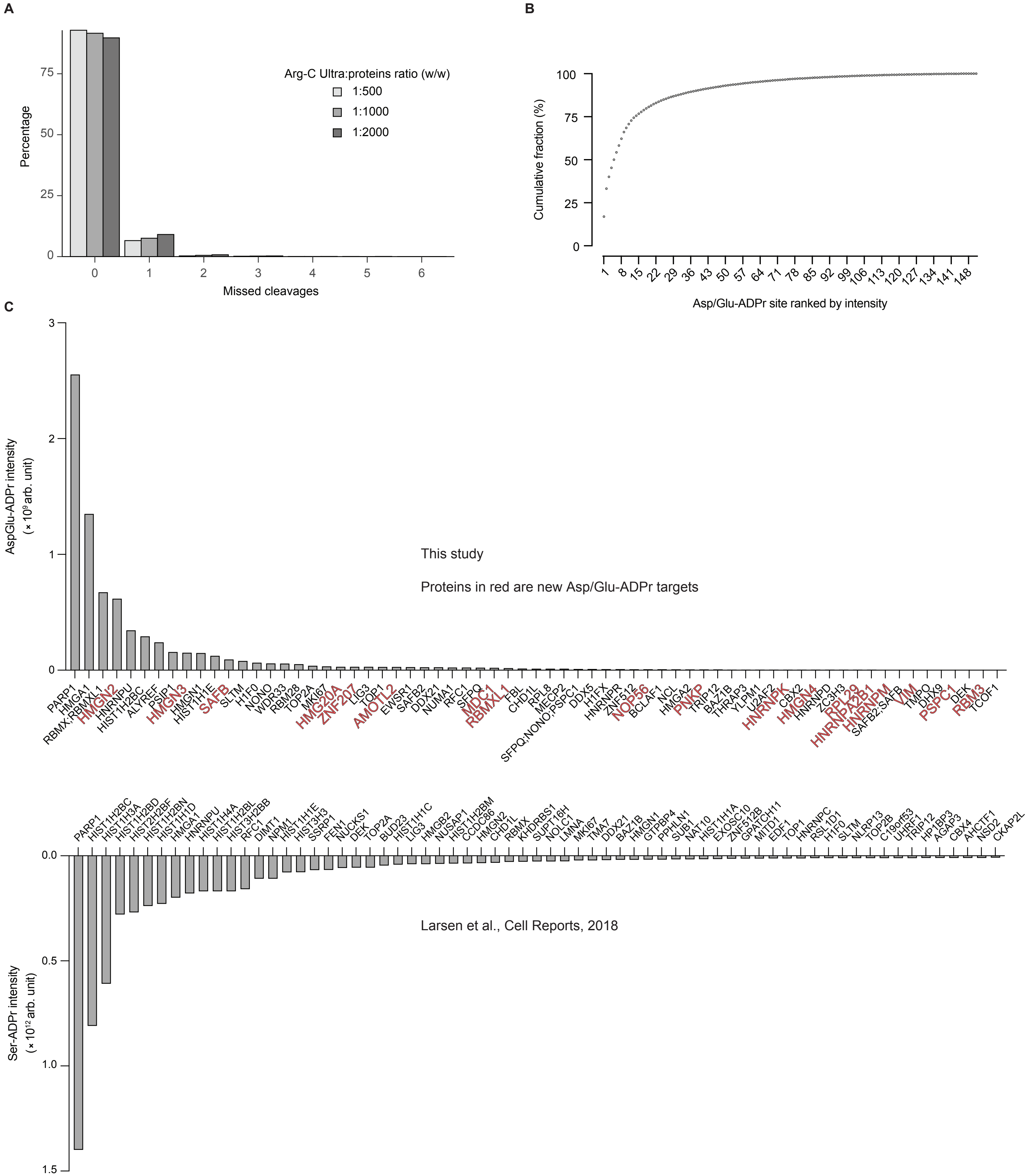
A
Percentage
75
50
25
0
Arg-C Ultra:proteins ratio (w/w)
1:500
1:1000
1:2000
0 1 2 3 4 5 6
Missed cleavages
B
Cumulative fraction (%)
100
75
50
25
0
1 8 15 22 29 36 43 50 57 64 71 78 85 92 99 106 113 120 127 134 141 148
Asp/Glu-ADPr site ranked by intensity
C
AspGlu-ADPr intensity
(× 10⁹ arb. unit)
3
2
1
0
This study
Proteins in red are new Asp/Glu-ADPr targets
PARP1 HMGA1 RBMX;RBMXL1 HMGN2 HNRNPU HIST1H2BC ALYREF PSIP1 HMGN3 HMGN1 HIST1H1E SAFB SLTM H1F0 NONO WDR33 RBM28 TOP2A MKI67 HMG20A ZNF207 LIG3 TOP1 AMOTL2 EWSR1 SAFB2 DDX21 NUMA1 RFC1 SFPQ MDC1 RBMXL1 FBL CHD1L RPL8 MECP2 SFPQ;NONO;PSPC1 DDX5 H1FX HNRNPR ZNF512 NOP56 BCLAF1 NCL HMGA2 PNKP TRIP12 BAZ1B THRAP3 YLPM1 U2AF2 HNRNPK CBX2 HMGN4 HNRNPD ZC3H3 RPL29 HNRNPA2B1 HNRNPM SAFB2;SAFB VIM TMPO DHX9 PSPC1 DEK RBM3 TCOF1
Ser-ADPr intensity
(× 10¹² arb. unit)
0.0
0.5
1.0
1.5
PARP1 HIST1H2BC HIST1H3A HIST1H2BD HIST2H2BF HIST1H2BN HIST1H1D HMGA1 HNRNPU HIST1H4A HIST1H2BL HIST3H2BB RFC1 DIMT1 NPM1 HIST1H1E HIST3H3 SSRP1 FEN1 NUCKS1 DEK TOP2A BUD23 HIST1H1C LIG3 HMGB2 NUSAP1 HIST1H2BM CCDC86 HMGN2 CHD1L RBMX KHDRBS1 SUPT16H NOLC1 LMNA MKI67 TMA7 DDX21 BAZ1B HMGN1 GTPBP4 PPHLN1 SUB1 NAT10 HIST1H1A EXOSC10 ZNF512B GPATCH11 MTDH EDF1 TOP1 HNRNPC RSL1D1 H1F0 SLTM NLRP13 TOP2B C19orf53 UHRF1 TRIP12 HP1BP3 AGAP3 CBX4 AHCTF1 NSD2 CKAP2L
Larsen et al., Cell Reports, 2018

Supp. Fig. 4: Identification of mono-Asp/Glu-ADPr on additional targets through ester bond preservation

a, Percentage of missed cleavages from LC-MS/MS analyses of whole-cell lysates samples digested with varying ratios (w/w protease-to-protein ratio) of Arg-C Ultra for 3 h at 37 ˚C in acidic buffer (20 mM sodium acetate pH 5.1, 5 mM DTT). n = 3 independent experiments. **b,** Asp/Glu-ADPr sites identified in our study ranked by cumulative intensity. **c,** Top: complete list of mono-Asp/Glu-ADP-ribosylated proteins identified in our study, ranked by the cumulative intensity of the ADPr sites. The proteins highlighted in red have not been previously identified as Asp/Glu-ADPr targets in large-scale ADPr studies. Bottom: data derived from Larsen et al., Cell Reports, 2018, list of top Ser-ADP-ribosylated proteins ranked by cumulative intensity. Most Asp/Glu-ADPr targets are also Ser-ADPr targets. The data shown in b, c derives from the same experiments shown in Fig. 4.

A

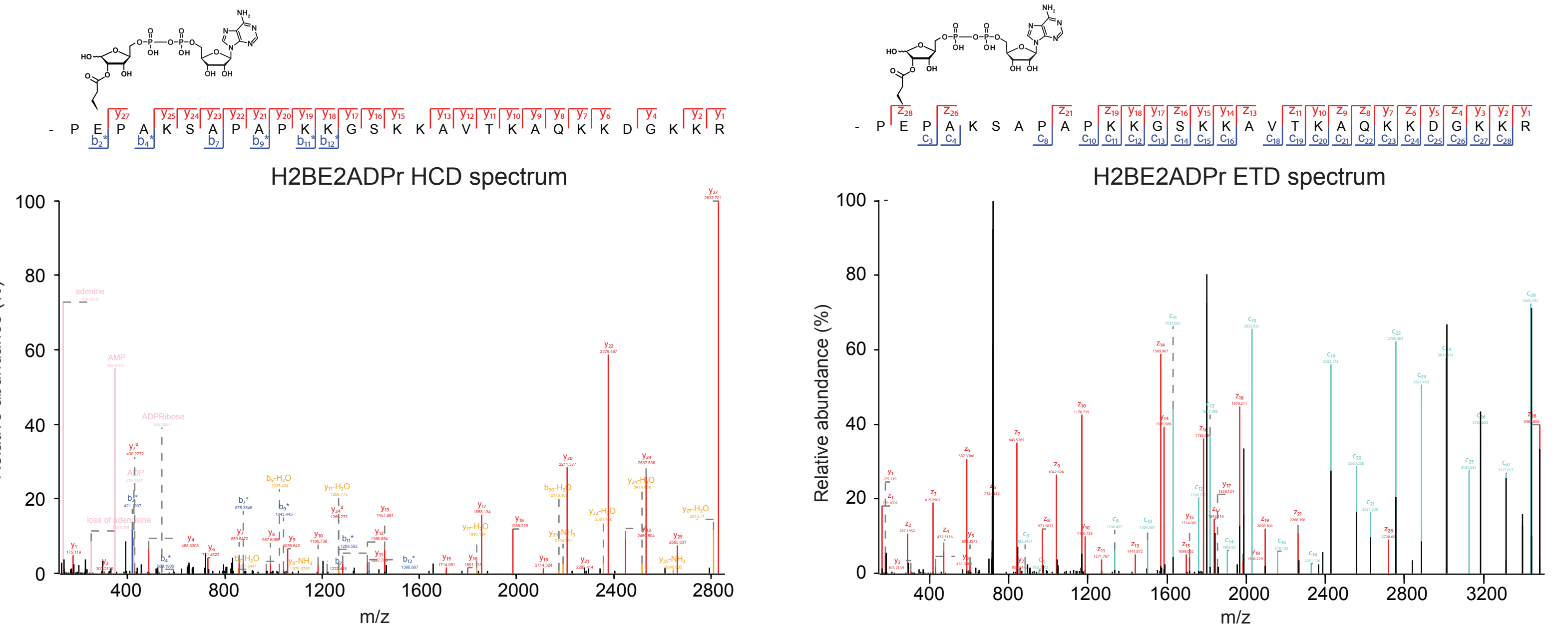
H2BE2ADPr HCD spectrum
H2BE2ADPr ETD spectrum
Relative abundance (%)
m/z

B

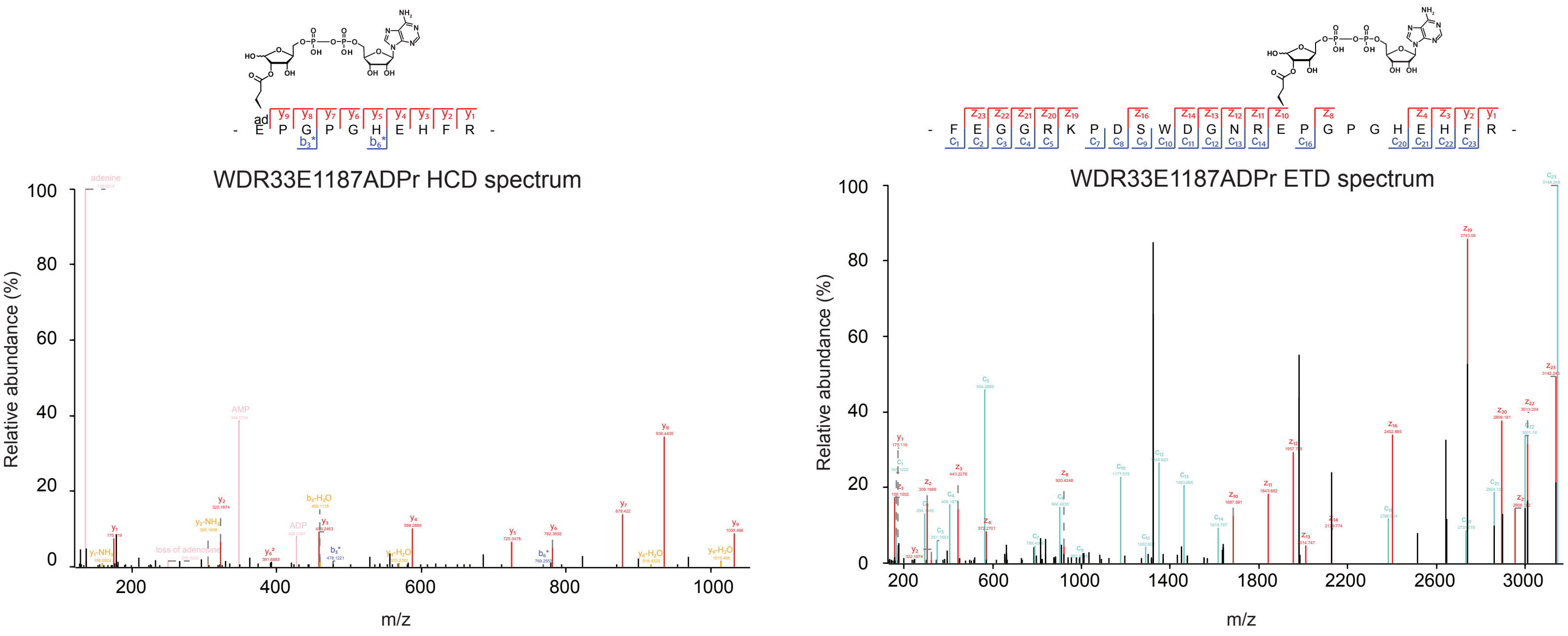
WDR33E1187ADPr HCD spectrum
WDR33E1187ADPr ETD spectrum
Relative abundance (%)
m/z

C

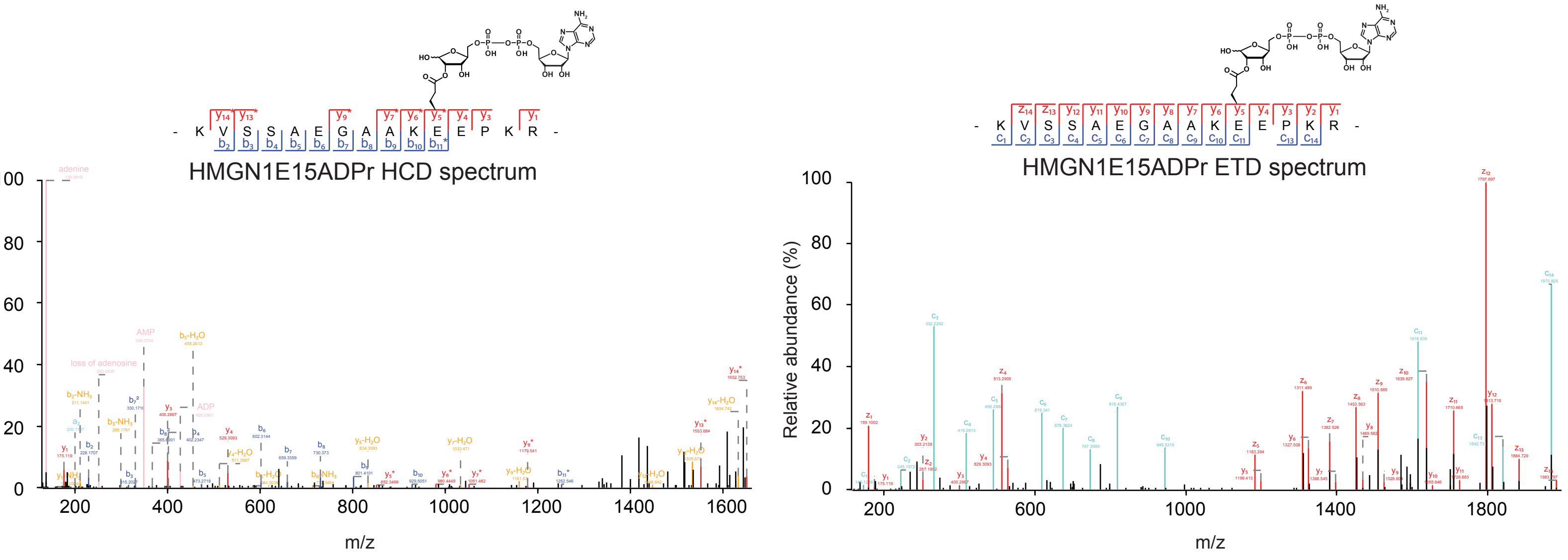
HMGN1E15ADPr HCD spectrum
HMGN1E15ADPr ETD spectrum
Relative abundance (%)
m/z

Supp. Fig. 5: ETD and HCD spectra of ADP-ribosylated peptides identified upon mono-ADPr enrichment

a-c, Left: high-resolution High-energy Collisional Dissociation (HCD) spectra; right: high-resolution Electron-Transfer Dissociation (ETD) spectra for **a,** H2BE2ADPr **b,** WDR33E1187ADPr **c,** HMGN1E15ADPr**.** HCD fragmentation of the Asp/Glu-ADPr peptide leads to loss of AMP (Δmass = - 347.06 Da) and leaves phosphoribose-H_2O (Δmass = 193.998) as a diagnostic fragment that can be used to identify the site of ADPribosylation. In ETD fragmentation the full ADP-ribose is preserved on the fragment ions (Δmass = + 541.062 Da). Data from the same experiments shown in Fig. 4.

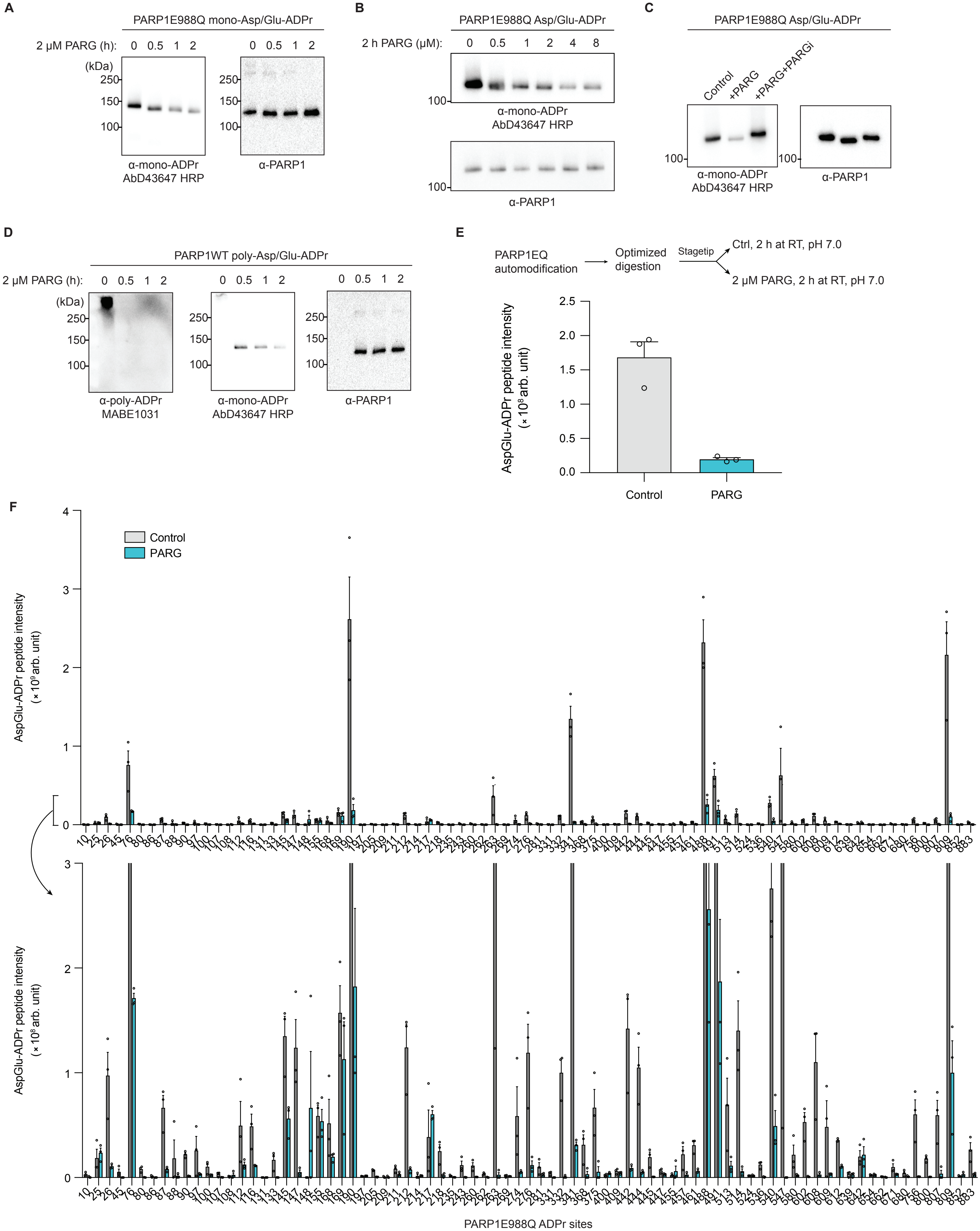

A
PARP1E988Q mono-Asp/Glu-ADPr
2 µM PARG (h): 0 0.5 1 2
(kDa)
250
150
100
α-mono-ADPr
AbD43647 HRP
α-PARP1
B
PARP1E988Q Asp/Glu-ADPr
2 h PARG (µM): 0 0.5 1 2 4 8
100
α-mono-ADPr
AbD43647 HRP
α-PARP1
C
PARP1E988Q Asp/Glu-ADPr
Control
+PARG
+PARG+PARGi
100
α-mono-ADPr
AbD43647 HRP
α-PARP1
D
PARP1WT poly-Asp/Glu-ADPr
2 µM PARG (h): 0 0.5 1 2
(kDa)
250
150
100
α-poly-ADPr
MABE1031
α-mono-ADPr
AbD43647 HRP
α-PARP1
E
PARP1EQ automodification
Optimized digestion
Stagetip
Ctrl, 2 h at RT, pH 7.0
2 µM PARG, 2 h at RT, pH 7.0
AspGlu-ADPr peptide intensity
(× 10⁸ arb. unit)
Control
PARG
F
Control
PARG
AspGlu-ADPr peptide intensity
(× 10⁹ arb. unit)
AspGlu-ADPr peptide intensity
(× 10⁸ arb. unit)
10 25 26 45 76 80 86 87 88 90 97 100 107 108 112 116 131 133 145 147 148 155 168 169 190 197 205 209 211 212 214 217 218 235 243 260 262 263 269 274 276 281 331 332 341 368 375 400 409 442 444 445 447 455 457 461 488 491 513 514 524 536 540 547 580 602 608 609 612 639 642 654 662 671 680 756 800 807 809 852 883
PARP1E988Q ADPr sites

Supp. Fig. 6: Human PARG can hydrolyse mono-Asp/Glu-ADPr in cells.

a, Automodified PARP1E988Q was incubated with Olaparib and 2 µM recombinant PARG for the indicated times, then subjected to immunoblotting with the indicated antibodies. **b,** Automodified PARP1E988Q was incubated with recombinant PARG for 2 h at the indicated concentration, then subjected to immunoblotting with the indicated antibodies. **c,** Automodified PARP1E988Q was incubated with 2 µM recombinant PARG for 2 h with or without PARGi (PDD00017273, Sigma), then subjected to immunoblotting with the indicated antibodies. **d,** Poly-Asp/Glu-ADPribosylated PARP1 (WT) was incubated with Olaparib and 2 µM PARG for the indicated times, then subjected to immunoblotting with the indicated antibodies. **e,** Top: schematic of the experimental setup. PARP1E988Q was automodified, then digested according to the optimized digestion protocol. The resulting peptides were stagetipped, then treated with or without 0.5 µM recombinant PARG for 2 h at room temp. PARG treatment of intact PARP1E988Q, before digestion, would lead to large amounts of PARG peptides upon digestion, which would significantly compromise the analysis of ADPr peptides. Bottom: average ADPr intensity ± SEM for the peptides identified in the ctrl and PARG-treated samples. See also related Supplementary Fig. 6f. n = 3 independent experiments shown as individual dots. **f,** Same experiment as in Supplementary Fig. 6e, showing the intensity ± SEM for each high-confidence identified ADPr site in ctrl and PARG treatment samples. Bottom: zoomed-in plot in the intensity range 0 to $3e^8$ to visualize low-abundance sites. n = 3 independent experiments shown as individual dots. For all experiments **a-f,** to control for chemical hydrolysis of Asp/Glu-ADPr both control and PARG-treated samples were incubated together for the same total processing time. Shown is a representative of three independent replicates.

A

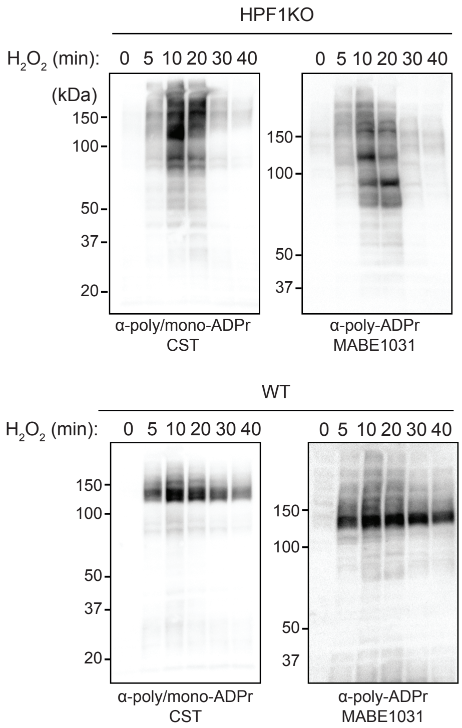

HPF1KO
H_2O_2 (min): 0 5 10 20 30 40
0 5 10 20 30 40
(kDa)
150
100
50
37
20
150
100
50
37
α-poly/mono-ADPr
CST
α-poly-ADPr
MABE1031
WT
H_2O_2 (min): 0 5 10 20 30 40
0 5 10 20 30 40
150
100
50
37
20
150
100
50
37
α-poly/mono-ADPr
CST
α-poly-ADPr
MABE1031

Supp. Fig. 7: Mono-Asp/Glu-ADPr belongs to the initial wave of PARP1 signaling.

a, Related to Figure 7a, the same samples were also stained with antibodies against poly/mono-ADPr (E6F6A, Cell Signaling Technology) and poly-ADPr (MABE1031, Millipore).
